# Hemodialysis Tends to Improve Thyroid Function by Restoring Hormone Levels in ESRD Patients Compared to Non-Dialysis Kidney Disease Patients: A Case–Control Study

**DOI:** 10.3390/diseases14040128

**Published:** 2026-04-01

**Authors:** Hasibul Islam, Shahad Saif Khandker, Anwara Khatun, Ehsan Suez, Alif Hasan Pranto, Dewan Zubaer Islam, Rahima Begum, Md. Nizam Uddin, Md. Ashraful Hasan, Md. Shah Alam, A. N. M. Mamun-Or-Rashid

**Affiliations:** 1 Department of Biochemistry and Molecular Biology, Jahangirnagar University, Dhaka 1342, Bangladesh; 2 Department of Biochemistry and Molecular Biology, Gono Bishwabidyalay, Dhaka 1344, Bangladesh; 3 Department of Microbiology, Gono Bishwabidyalay, Dhaka 1344, Bangladesh; 4 Institute of Bioinformatics, University of Georgia, Athens, GA 30602, USA; 5 School of Pharmacy, Brac University, Dhaka 1212, Bangladesh; 6 Department of Microbiology, Jahangirnagar University, Dhaka 1342, Bangladesh; 7 Department of Microbiology, Gonoshasthaya Samaj Vittik Medical College, Dhaka 1344, Bangladesh; 8 Department of Environmental and Occupational Health, University of Pittsburgh, Pittsburgh, PA 15261, USA

**Keywords:** dialysis, thyroid, hormone, ESRD, CKD, Bangladesh, case–control, triiodothyronine, thyroxine, TSH

## Abstract

Background: Chronic kidney disease (CKD) represents an escalating global health burden, fundamentally altering morbidity and mortality trajectories across the world, particularly as it advances into end-stage renal disease (ESRD). Beyond the primary decline in renal filtration and excretion, a wide spectrum of endocrine and metabolic derangements frequently accompanies kidney failure, with thyroid dysfunction emerging as a critical complication. Methods: The current study was designed to rigorously evaluate the nuanced association between thyroid hormone dynamics—specifically thyrotropin (TSH), triiodothyronine (T3), and thyroxine (T4)—and renal status in three distinct cohorts: individuals with suspected thyroid issues but normal renal function (NPs), non-dialysis kidney disease patients (NDKPs), and patients undergoing maintenance hemodialysis (DPs). Data were collected from a clinical setting in Bangladesh, involving 161 subjects. Results: The results demonstrated that patients in the DP cohort exhibited slightly elevated thyroid hormone levels relative to those in the NDKP cohort. Specifically, within the subgroups of patients exhibiting normal or sub-reference hormonal levels, dialysis patients maintained higher concentrations than their non-dialysis counterparts. Demographic stratification further revealed that males, females, and individuals younger than 45 years were more likely to demonstrate restorative hormonal profiles in the DP group than in the NDKP group. Conclusions: These collective outcomes suggest that renal replacement therapy, specifically hemodialysis, may serve to stabilize or improve thyroid function in ESRD patients by potentially mitigating the suppressive effects of uremic toxins and normalizing homeostatic feedback loops.

## 1. Introduction

Chronic kidney disease (CKD) is a significant global health challenge, contributing substantially to morbidity and mortality in both developed and developing countries, particularly when it progresses to end-stage renal disease (ESRD) [1,2]. The global prevalence of CKD is estimated at approximately 13.4%, and 4.9 to 7.1 million individuals with ESRD require renal replacement therapy [3,4]. Thyroid hormones play a crucial role in the physical and cognitive development of children and adults [5]. These hormones are vital for supporting the optimal functioning of all major organs, including the kidneys, thereby helping to preserve overall homeostasis and essential bodily processes; consequently, any complications arising from thyroid hormone imbalances can significantly disrupt normal physiological functions [6,7]. 

Thyroid hormones such as triiodothyronine (T3) and thyroxine (T4) are produced by the thyroid gland [8]. The thyroid gland primarily produces T4, which accounts for approximately 80% of thyroid hormone production, and T3, which accounts for about 20%. While all T4 in the bloodstream originates in the thyroid gland, only 20% of circulating T3 is directly produced by the thyroid; the remaining 80% is converted from T4 outside the thyroid (extrathyroidally) [9,10]. This process underscores the intricate dynamics of thyroid hormone synthesis and conversion, as discussed in studies on thyroid function assessment and thyroid disorders in chronic kidney disease [11]. Thyroid-stimulating hormone (TSH), secreted by the pituitary gland, is crucial for normal thyroid function [12]. TSH production is regulated by a hypothalamic hormone, thyrotropin-releasing hormone (TRH), which is influenced by environmental, developmental, and daily rhythms [13,14]. The regulation of thyroid hormone secretion is achieved through feedback inhibition by the hypothalamic–pituitary–thyroid axis (HPT). Initially, the hypothalamus secretes TRH, which then travels to the anterior pituitary gland and stimulates the synthesis of TSH [15].

T3 and T4 play crucial roles in regulating metabolism, energy production, and overall growth and development [16,17]. Elevated TSH levels often signal conditions such as hypothyroidism and Hashimoto’s thyroiditis [12]. The kidneys play a crucial role in thyroid hormone metabolism, breakdown, and elimination; consequently, renal impairment significantly alters thyroid physiology [18]. Nevertheless, thyroid hormones are essential for regulating the glomerular filtration rate (GFR) by controlling renal blood flow [6].

Previous studies indicate that thyroid comorbidities are more prevalent in individuals with CKD compared to the general population [16,19]. Endocrine disorders were found to be highly prevalent among patients with CKD, especially those undergoing dialysis. CKD affects multiple endocrine and metabolic processes [3,20]. In addition, inflammation was reported to affect and develop both CKD and thyroid dysfunction, which indicates the correlation of CKD and thyroid complications [21,22]. Conversely, a previous study reported that hemodialysis induced a significant rise in thyroid hormone levels among ESRD patients [23].

Therefore, the objective of this cohort study was to investigate the association between and compare the levels of T3, T4, and TSH in patients undergoing renal replacement therapy (hemodialysis), patients not receiving dialysis, and patients suspected of having thyroid complications only, with no other confirmed kidney or other complications.

## 2. Materials and Methods

### 2.1. Ethical Approval, Study Settings, and Participants

The study was conducted from December 2024 to August 2025 in the hospital settings of Gonoshasthaya Nagar Hospital, Dhaka, Bangladesh. Ethical approval was obtained from the Center for Multidisciplinary Research, Gono Bishwabidyalay, Dhaka, Bangladesh (Registration no: CMR/ER/026).

### 2.2. Inclusion and Exclusion Criteria

Patients suspected of having thyroid complications and tested for T3, T4, and TSH were included in this study. The patients in this study cohort were categorized into the following three groups:

1. Normal patients (NPs): individuals suspected of having thyroid complications with no kidney complications or other confirmed diagnosed diseases.
2. Non-dialysis kidney disease patients (NDKPs): individuals with kidney or renal complications confirmed by a creatinine level > the reference range (0.6–1.3 mg/dL) who were suspected of having thyroid complications.
3. Dialysis patients (DPs): ESRD patients receiving dialysis for 1–2 years, but not less than 1 year, who were suspected of having thyroid complications.

Patients who did not have, or were not suspected of having, thyroid complications, or who were taking medication or treatment associated with thyroid complications, or had other chronic illnesses or confirmed diagnosed comorbidities such as diabetes or hypertension, were excluded from this study.

### 2.3. Sample Collection

Blood samples from DPs were collected 5–6 h before each session through the fistula. For NPs and NDKPs, blood was drawn by venipuncture. The serum was separated from the hematocrit by centrifugation. If the test was not performed immediately after separation, the serum was stored at −20 °C.

### 2.4. Measurement of T3, T4, and TSH Levels

All hormones, including triiodothyronine (T3), thyroxine (T4), and thyrotropin (TSH), were quantified using a colorimetric enzyme-linked immunosorbent assay (ELISA). Total triiodothyronine (T3) levels were measured with the Biorex Diagnostics TT3 kit (BXE0701A, Antrim, UK), with a reference range of 0.8 to 2.0 ng/mL. Total thyroxine (T4) concentrations were assessed with the Biorex Diagnostics TT4 kit (BXE0711A, UK), with reference values ranging approximately from 4.5 to 12.0 µg/dL, where for females and males the ranges were 4.8 to 11.6 µg/dL and 4.4 to 10 µg/dL, respectively. Thyrotropin (TSH) levels in human serum were quantified with a colorimetric ELISA using the Biorex Diagnostics TSH kit (BXE0681A, UK), with a reference range of 0.4 to 4.0 µIU/mL. As per the test procedure, the reference range of the T3 and TSH was the same for males and females. However, for T4, the reference range was different for males and females. The optical density (OD) of all analyzed hormones (T3, T4, and TSH) was measured at 450 nm, a wavelength commonly used in ELISA procedures to accurately quantify thyroid hormone concentrations in biological samples. Measurements were conducted with a Multiskan FC microplate photometer (Multiskan FC, Thermofisher Scientific, Waltham, MA, USA).

### 2.5. Statistical Analysis

Statistical analyses were conducted using SPSS (version 16.0), and data assembly, general calculations, percentage determinations, and graphical presentations were performed using Microsoft Excel (2010). Mean, standard deviation, Pearson correlation, and *p*-value were assessed for the statistical analysis.

## 3. Results

### 3.1. Participant Demographics and Hormonal Level Determination

The study included 161 patients, divided into three groups: 106 NP, 32 NDKP, and 23 DP. The mean age of the participants was recorded as 42.49 ± 17.89 years. Detailed demographic information is provided in Table 1.

The distribution of patients exhibiting normal (within reference values), elevated (>reference values), and diminished (<reference values) hormone levels is presented in Table 2.

### 3.2. Comparison of Thyroid Hormone Levels

**Figure 1:**
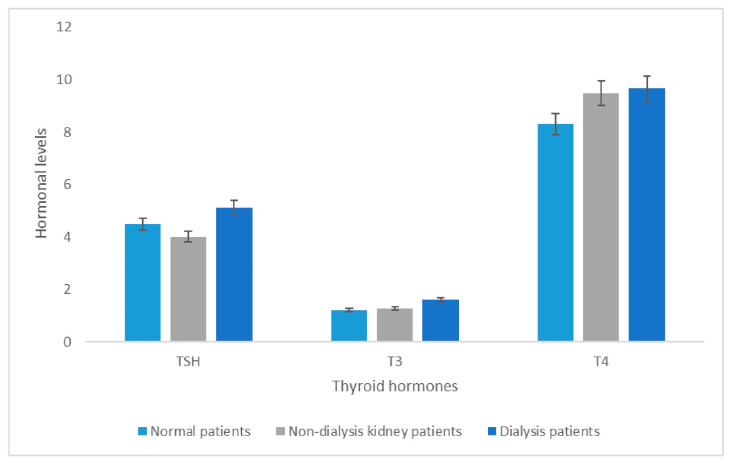
Comparison of mean hormonal levels of thyroid hormones (i.e., TSH, T3, and T4) among normal (NP), non-dialysis kidney (NDKP), and dialysis (DP) patients.

A comparative analysis of TSH, T3, and T4 across the three groups indicated that the DP group exhibited the highest levels of all three hormones. Notably, TSH levels in the NP group were significantly elevated compared to those in the NDKP group. Conversely, T3 levels were slightly higher in the NDKP group than in the NP group. Analogous trends were observed in T4 levels, with NP and NDKP showing results similar to those of T3 (Figure 1).

**Figure 2:**
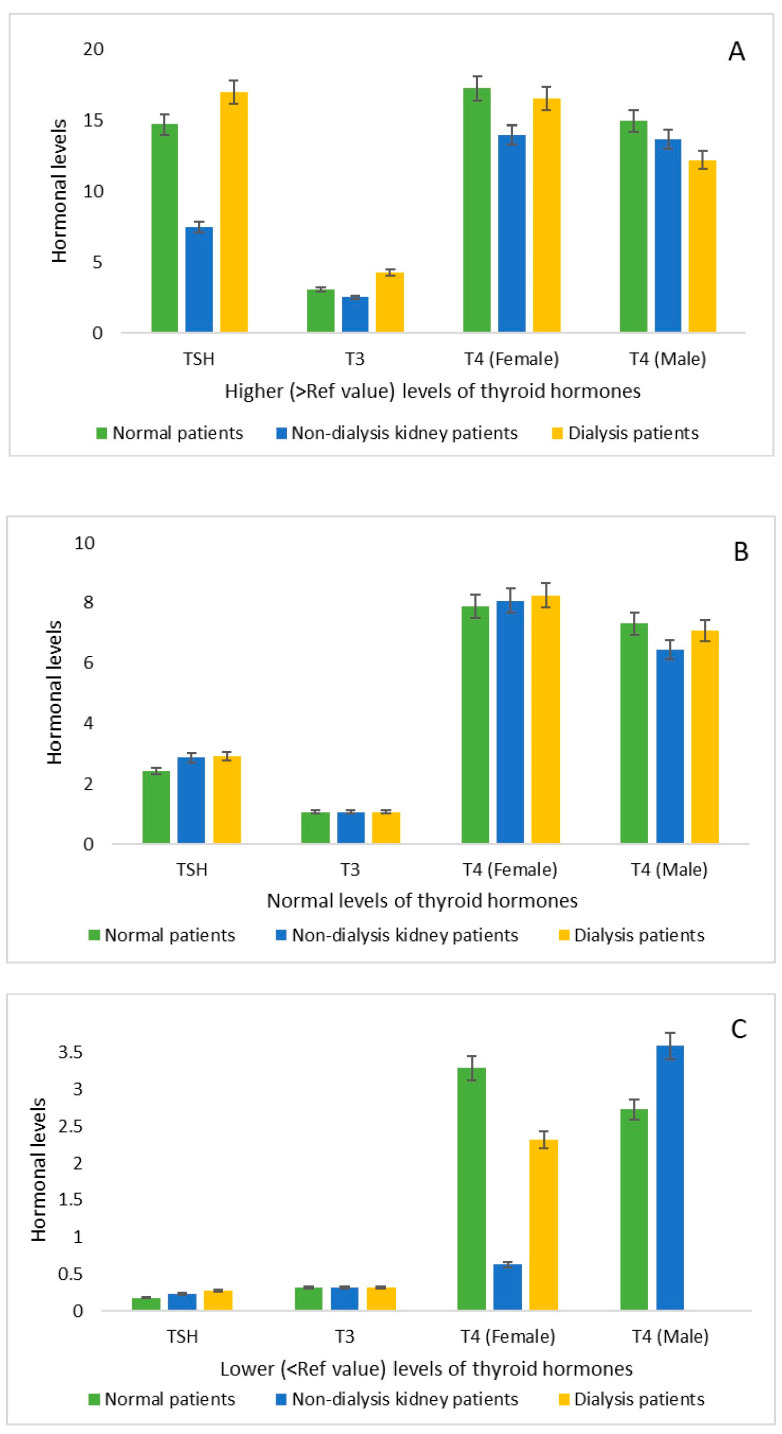
Comparisons among the thyroid hormones (i.e., TSH, T3, and T4) based on (**A**) higher levels (>Ref value), (**B**) normal levels (within the Ref value), and (**C**) lower levels (<Ref value) among normal, non-dialysis kidney disease, and dialysis patients. The TSH, T3, and T4 units were µIU/mL, ng/mL, and (µg/dL), respectively.

Further examination of hormonal levels across lower, normal, and higher categories revealed that, for elevated hormonal levels, TSH, T3, and T4 were substantially higher in the DP group than in NDKP, except for T4 levels among male patients. Similar patterns were observed for normal hormone levels in TSH and T4, although T3 levels remained consistent across all groups. When focusing solely on lower hormone levels, T3 levels were equivalent between DP and NDKP, whereas TSH and T4 (in females) were elevated in DP compared to NDKP. Moreover, no instances of low T4 levels were identified among males (Figure 2).

### 3.3. Comparison of Hormone Levels Based on Sex and Age

Hormonal levels were analyzed with respect to sex and age. In male participants, both TSH and T4 levels were highest in the DP group compared with the NP and NDKP groups. Among female participants, all three hormones showed elevated levels in the DP group compared with the other two groups (Table 3).

In terms of age stratification, TSH and T3 levels were higher in the DP group than in the other groups. Conversely, the T4 levels in the NDKP group exceeded those in both the NP and DP groups among patients aged <45 years. In the cohort of patients over 45, T4 levels were generally higher in the NP group than in the other two groups, except in the DP group.

### 3.4. Association Between Hormone Levels

**Figure 3:**
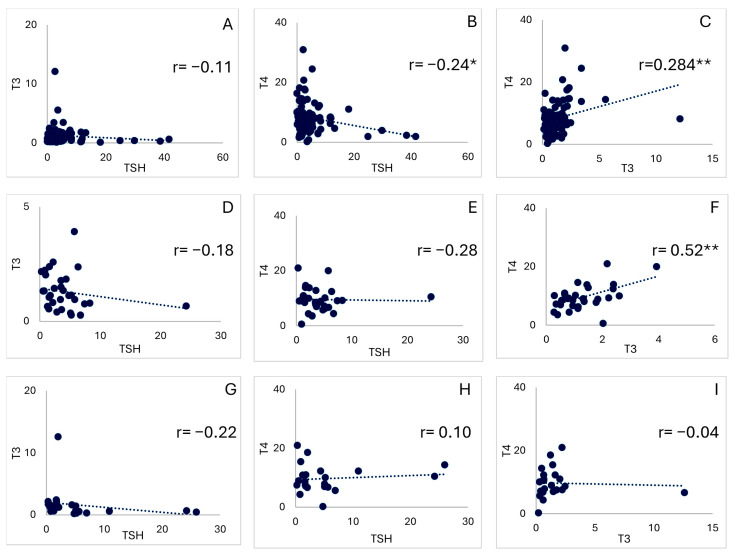
Association of thyroid hormones (i.e., TSH, T3, and T4) among NPs, NDKPs, and DPs. The plot shows the association between the levels of (**A**) TSH and T3, (**B**) TSH and T4, (**C**) and T3 and T4 in NPs; (**D**) TSH and T3, (**E**) TSH and T4, (**F**) and T3 and T4 in NDKPs; and (**G**) TSH and T3, (**H**) TSH and T4, (**I**) and T3 and T4 in DPs. * denotes a significant correlation at the 0.05 level, and ** denotes a significant correlation at the 0.01 level. The TSH, T3, and T4 units were µIU/mL, ng/mL, and µg/dL, respectively.

The assessment of correlations between pairs of hormones (TSH, T3, and T4) across all three groups revealed a negative correlation between TSH and T3. A positive correlation between TSH and T4 was observed in the DP group, whereas a negative correlation was observed in the NP and NDKP groups. Furthermore, negative correlations between T3 and T4 were observed exclusively in the DP group, whereas positive correlations were significantly established in the other two groups. Notably, the negative correlation between TSH and T4 was statistically significant in the NP group (Figure 3).

## 4. Discussion

Thyroid autoimmunity and subclinical primary hypothyroidism are prevalent in CKD patients who do not require long-term dialysis [24]. In a study, 23.1% of CKD patients with a GFR below 30 mL/min/1.73 m^2^ were found to have hypothyroidism. Furthermore, it has been reported that 18–20% of CKD patients not needing renal replacement therapy experience either subclinical or clinically apparent hypothyroidism [25].

The kidneys play a crucial role in iodine clearance, predominantly via glomerular filtration [26,27]. In patients with CKD, reduced GFR decreases iodine clearance, leading to higher plasma iodide levels, which eventually result in increased iodide uptake by thyroid tissue [28,29]. Excess iodide may inhibit thyroid hormone production by disrupting the pituitary–thyroid axis, a phenomenon known as the Wolff–Chaikoff effect. These physiological alterations provide a plausible explanation for the higher incidence of hypothyroidism observed in CKD patients [30]. Both subclinical and overt hypothyroidism are common in CKD patients, indicating a prevalence of diminished thyroid function, whether symptomatic or asymptomatic [31]. Our present study indicates that patients with kidney disease (NDKPs) had lower TSH levels than patients without kidney disease (NPs). Interestingly, patients receiving regular hemodialysis (DPs) had higher hormonal levels for all three evaluated thyroid hormones (i.e., TSH, T3, and T4) (Figure 1). While analyzing the higher, normal, and lower hormonal levels other than normal T4 (males), the DP group was found to have higher hormonal levels as compared to the NDKP group, and no cases were found in the T4 (males) within the category of lower hormonal levels (<reference value) (Figure 2). Previously, a study by Sanai et al. reported rising serum free T3 and T4 levels within the control or normal reference range after hemodialysis, especially among ESRD patients with chronic glomerulonephritis [32]. Findings from our study also align with those of a study by Shamsadini et al., which reported that hemodialysis restored serum levels of thyroid hormone to normal ranges among patients with chronic renal failure [33]. During participant selection, it was found that the majority of the patients were female, which is supported by a study reporting that thyroid dysfunction occurs predominantly in females (Table 1) [34]. Therefore, the effect of dialysis was more pronounced in female patients than in male patients, with mean hormone levels (TSH, T3, and T4) higher in the DP group than in the other two groups (Table 3). Again, most patients in the cohort are close to 40 years of age, and in the NDKP and DP groups, the mean age was >40 years, which aligns with previous studies reporting that hyperthyroidism is quite common in older people as compared to the younger [35,36]. This phenomenon was observed by comparing the levels between age groups, as we observed that in the age group <45 years, dialysis did have an impact on elevating the hormone levels in most of the cases, whereas in the age group >45 years, dialysis did not have such an effect on the hormonal levels (Table 3).

In the association analysis, we observed a similar pattern between the NP and NDKP groups: both showed negative correlations between T3 and TSH and between T4 and TSH, whereas T3 and T4 showed a significant positive association. However, in the case of the DP group, we observed that T4 vs. TSH showed a slight positive correlation, and T3 vs. T4 showed an inverse correlation, both of which were not significant (Figure 3). A moderate inverse relationship between TSH and T4 was determined in a previous study among patients with primary thyroid failure [37,38]. In addition, T4 primarily serves as a prohormone reservoir that is converted to the more biologically active T3 in tissues, making T3 pivotal in mediating metabolic processes, while T4 ensures a steady supply necessary for physiological homeostasis, which indicates that, due to regular dialysis, T4 levels and activity were slightly higher in the DP group than in the T3 group [39,40]. Overall, our findings suggest that regular dialysis moderately improves thyroid hormone levels in kidney disease patients, especially compared with those not on dialysis (Table 4). Further research is required to elucidate the underlying molecular mechanisms governing these observed changes.

## 5. Limitations of This Study

Including additional participants in this study, particularly in the NDKP and DP groups, was not feasible. Furthermore, the absence of a longitudinal analysis involving a larger cohort represents a notable limitation of the research. Due to the small sample size, we also could not do any interaction modeling. In addition, we could not include other renal, nutritional, or inflammatory biomarkers, which is another limitation of this study. Again, as the study was conducted at a single center, the findings of the study may not generalize to other ethnic populations, regions with different iodine status, healthcare systems with different dialysis protocols, and populations with differing nutritional or inflammatory profiles, which we acknowledge as a limitation of this study.

## 6. Future Perspectives and Recommendations

- Research Gap: Limited research exists on the interplay among thyroid function, hormone levels, and dialysis outcomes in CKD patients, especially in the Bangladeshi context.
- Future Research Direction: Multifactorial longitudinal studies with multicenter designs and larger and more diverse cohorts are needed to robustly evaluate the interplay among thyroid function, hormone levels, and dialysis outcomes in CKD patients.
- Study Design Justification: Such studies are crucial to enhance generalizability, mitigate selection bias, and enable comprehensive statistical analysis.

## 7. Conclusions

Dialysis has been shown to moderately enhance thyroid hormone levels and activity in patients with renal complications compared to those with kidney disease who are not undergoing dialysis. These findings indicate that routine dialysis may confer potential benefits for individuals with chronic kidney disease (CKD) or end-stage renal disease (ESRD). Further investigation involving larger cohorts of dialysis and non-dialysis patients, stratified by hypo- and hyperthyroid conditions, is warranted to elucidate these observations more comprehensively.

## Figures and Tables

**Table 1:** Participant demographics and overall levels of thyroid hormones among each group.

	NPs	NDKPs	DPs
Participant demographics			
Overall, patients, *n*	106	32	23
Age in years (mean ± SD)	38.64 ± 17.05	43.75 ± 17.54	43.00 ± 17.51
Male, *n*	32	10	8
Age in years (mean ± SD)	37.55 ± 17.01	45.7 ± 18.22	45 ± 15.90
Female, *n*	74	22	15
Age in years (mean ± SD)	39.12 ± 17.10	42.86 ± 17.53	41.93 ± 17.51
Overall levels of thyroid hormones			
TSH, µIU/mL (mean ± SD)	4.49 ± 0.24	4.01 ± 0.20	5.13 ± 0.25
T3, ng/mL (mean ± SD)	1.23 ± 0.06	1.29 ± 0.06	1.61 ± 0.08
T4, µg/dL (mean ± SD)	8.31 ± 0.41	9.49 ± 0.47	9.67 ± 0.48

Here, NPs: normal patients; NDKPs: non-dialysis kidney disease patients; DPs: dialysis patients.

**Table 2:** Participants (number and percentage) identified as having normal, high, and low levels of TSH, T3, and T4.

Patients of Different Groups, *n* (%)	Normal Level, *n* (%)	Higher Level, *n* (%)	Lower Level, *n* (%)
**NPs, *n* = 106 (100)**			
TSH	80 (75.47)	19 (17.92)	7 (6.60)
T3	67 (63.20)	17 (16.03)	22 (20.75)
T4 (female)	43 (40.56)	11 (10.37)	18 (16.98)
T4 (male)	19 (17.92)	9 (8.49)	6 (5.66)
T4 overall	62 (58.49)	20 (18.86)	24 (22.64)
**NDKPs, *n* = 32 (100)**			
TSH	26 (81.25)	5 (15.62)	1 (3.12)
T3	20 (62.50)	7 (21.32)	5 (15.62)
T4 (female)	17 (53.12)	5 (15.62)	1 (3.12)
T4 (male)	4 (12.5)	4 (12.5)	1 (3.12)
T4 overall	21 (65.62)	9 (28.12)	2 (6.25)
**DPs, *n* = 23 (100)**			
TSH	17 (73.91)	4 (17.39)	2 (8.69)
T3	13 (56.52)	5 (21.73)	5 (21.73)
T4 (female)	9 (39.13)	4 (17.39)	2 (8.69)
T4 (male)	4 (17.39)	4 (17.39)	0 (0.0)
T4 overall	13 (56.52)	8 (34.78)	2 (8.69)

Notes: Male, female, and overall patients with different T4 levels are presented separately, as the reference values for males and females differed. Here, NPs: normal patients; NDKPs: non-dialysis kidney patients; DPs: dialysis patients.

**Table 3:** The levels of thyroid hormones (i.e., TSH, T3, and T4) among normal, non-dialysis kidney disease, and dialysis patients, stratified by sex and age category.

Category	Thyroid Hormones	Hormonal Levels (Mean)		
		NPs	NDKPs	DPs
Male	TSH	3.97	4.10	4.26
	T3	1.54	1.39	1.13
	T4	8.2	9.33	9.65
Female	TSH	4.71	3.96	5.60
	T3	1.10	1.24	1.86
	T4	8.36	9.55	9.67
Age < 45 y	TSH	3.94	4.64	5.54
	T3	1.24	1.40	2.32
	T4	8.59	11.07	8.74
Age > 45 y	TSH	5.89	3.25	4.64
	T3	1.24	1.23	1.12
	T4	8.11	7.95	10.77

Here, NPs: normal patients; NDKPs: non-dialysis kidney disease patients; DPs: dialysis patients; y: years.

**Table 4:** Summary of implications of thyroid hormone levels in kidney disease patients.

Patient Group	Thyroid Hormone Levels/Activity	Implications
Dialysis patients with renal complications	Moderately enhanced thyroid hormone levels and activity	Suggests potential benefits of routine dialysis for CKD/ESRD patients
Non-dialysis patients with kidney disease	Not significantly enhanced thyroid hormone levels and activity	Supports the need for further investigation with larger cohorts

## Data Availability

All data underlying the findings in our study are freely available in the manuscript. Further inquiries can be directed to the corresponding author.
